# Numerical Study on Entropy Generation of the Multi-Stage Centrifugal Pump

**DOI:** 10.3390/e24070923

**Published:** 2022-07-02

**Authors:** Baoxin Fan, Zhaoyu Liang, Ruonan Fan, Songying Chen

**Affiliations:** Key Laboratory of High-Efficiency and Clean Mechanical Manufacture, National Demonstration Center for Experimental Mechanical Engineering Education, School of Mechanical Engineering, Shandong University, Jinan 250061, China; 202034335@mail.sdu.edu.cn (B.F.); liangzhaoyu@mail.sdu.edu.cn (Z.L.); 202034334@mail.sdu.edu.cn (R.F.)

**Keywords:** multi-stage centrifugal pump, entropy generation, numerical analysis, energy loss

## Abstract

The energy loss of the multi-stage centrifugal pump was investigated by numerical analysis using the entropy generation method with the RNG k-ε turbulence model. Entropy generation due to time-averaged motion and velocity fluctuation was mainly considered. It was found that the entropy generation of guide vanes and impellers account for 71.2% and 23.3% of the total entropy generation under the designed flow condition. The guide vanes are the main hydraulic loss domains and their entropy generation is about 9 W/K, followed by impellers. There are vortices at the tongue of the guide vane inlet as well as flow separations in the impellers, which lead to entropy generation. The fluid impacts the outer surface of the guide vanes, resulting in the increase in entropy generation. There are refluxes near the guide vane tongues which also increase the entropy generation of this part. The entropy generation distribution of the guide vanes and impellers was investigated, which found that the positive guide vane has more entropy generation compared with the reverse guide. The entropy generation of the blade suction surface is higher compared with the pressure surface. This study indicated that the entropy generation method has distinct advantages in the assessment of hydraulic loss.

## 1. Introduction

Multi-stage centrifugal pumps are widely applied in industry fields, such as in power stations, refineries, petroleum extraction, transportation, and so on. With the increasing demand for pump applications, the traditional design scheme is not enough as it has a long design period and high cost, and it cannot clearly show the details of the internal flow. Therefore, new research schemes are needed. With the improvement of the computer, CFD technology is now widely applied to the fluid machinery [1,2].

**Figure 1:**
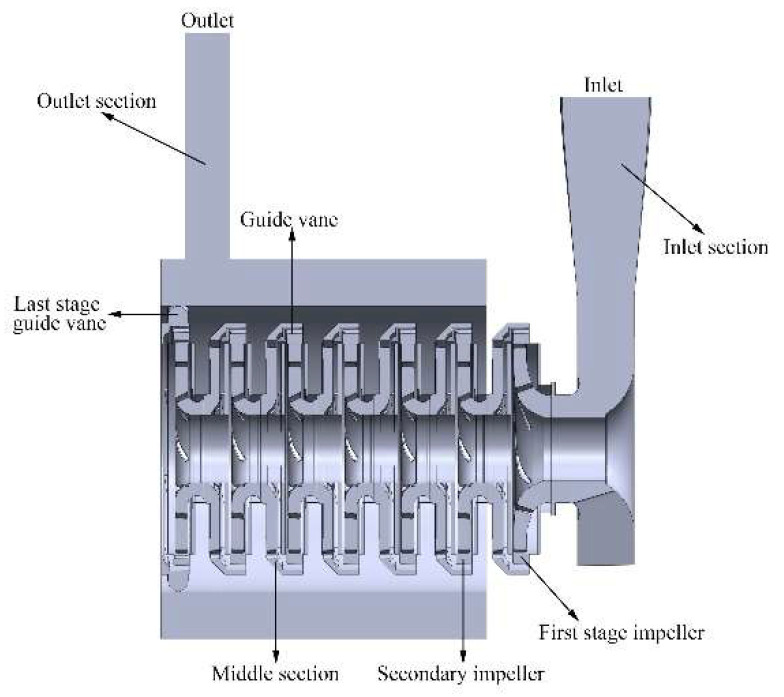
Internal structure of a BB5 multi-stage centrifugal pump.

Three-dimensional flow analysis in the pump is a basic means to improve the pump efficiency, predict the pump performance and analyze the energy loss. Figure 1 displays a BB5 multi-stage centrifugal pump that is composed of an inlet chamber, impellers, guide vanes, and shell. Due to the complexity of the multi-stage centrifugal pump structure and the rotation of impellers, there are many complicated flow phenomena in the pump such as secondary flow, recirculation flow, and boundary layer separation flow. Many researchers have used numerical simulation to study the flow field in the centrifugal pump. Guleren and Pinarbasi [3] analyzed the stalled flow of the centrifugal pump by the standard k-ε turbulence model. Jafarzadeh et al. [4] analyzed and compared the errors of the numerical results and experimental results in the centrifugal pump under different turbulence models. It was found that the results calculated with the RNG k-ε turbulence model are more accurate. Zhou et al. [5] used the S-A DES model and SST k-ω model to simulate and analyze the whole flow channel of the double-suction centrifugal pump, respectively. It resulted that the S-A DES model can capture more flow phenomena and it is more suitable to solve the unsteady flow. Feng et al. [6] analyzed the mechanism of the tip-clearance flow and the motion law of the gap vortices. It was found that the leakage flow in the tip clearance affects the mechanical fluid transfer and unsteady flow field in the impeller. Zhu et al. [7] used the RNG k-ε turbulence model and CFX software to calculate the Reynolds time-averaged N–S equations and studied the interaction and impact of the multi-stage centrifugal pump between various levels. 

However, it is not intuitive to analyze the flow information in fluid machinery only from the perspective of hydrodynamics. Due to the fluid viscosity and velocity fluctuation, the fluid mechanical energy is transformed into heat irreversibly. Entropy is a state parameter derived from the second law of thermodynamics and reflects the irreversibility of spontaneous processes. Bejan and Kestin [8] first put forwarded the concept of entropy generation calculation in the flow heat transfer process. Spurk [9] derived the transport equation of the entropy in detail. Kock and Herwig [10] treated the transport equation of entropy generation by the Reynolds-averaged process and classified entropy generation. Zhang et al. [11] defined the head loss coefficient by the method of entropy generation integration, which was verified by a conical diffuser case. Chu and Liu [12] studied the two-dimensional high-temperature limited jet and analyzed the primary causes of irreversible process loss by the entropy generation method. The research of entropy generation is a way to study the hydraulic loss and has been widely used in rotating machinery [13,14,15,16,17,18,19,20], which is meaningful to further optimize the structure and comprehensively explore the internal flow of the centrifugal pump. Li et al. [21] established a simplified hydraulic loss model by the entropy generation method and calculated the loss by three centrifugal pump models with different blade thickness. It was found that the calculated errors of the three models were close. Pei et al. [22] assessed the effect of the axial clearance between the impeller and diffuser cascade on energy loss in the pump by the generation method. It was found that turbulent dissipation is the primary reason causing hydraulic loss. Zhang et al. [23] assessed the energy consumption of a centrifugal pump by the local entropy generation method and accurately calculated the size and location of the hydraulic loss. It resulted that the local entropy generation calculation method is reliable. Li et al. [24] researched the head rupture characteristics that were caused by cavitation in the centrifugal pump. It was found that there is a certain correlation between entropy generation and the external characteristics of the centrifugal pump. Feng et al. [25] obtained a detailed distribution of the hydraulic loss and analyzed the distribution of energy loss of flow components during the power-off transition period in the centrifugal pump by the entropy generation method. It resulted that the energy loss of the centrifugal pump is related to the flow separation, backflow and vortex in the flow field. The internal structure and entropy generation of a multi-stage centrifugal pump are more complex than that of a general centrifugal pump. Koranteng Osman et al. [26] used the entropy generation term to evaluate the turbulent dissipation and to characterize the flow loss, studied the flow loss in a two-stage centrifugal pump, and verified the numerical results with experiments. Ren et al. [27] studied the influence of the guide ring on the energy loss of the multi-stage centrifugal pump by the entropy generation method. The calculated entropy generation rate value was in good agreement with the actual hydraulic loss value in the pump. Wu et al. [28] study the energy loss characteristics of a multi-stage pump with a different number of diffuser vanes by the entropy generation method. It was found that with the increase in the number of diffuser blades, the flow is more stable and the entropy generation decreases, but the intensity of pressure pulsation in the diffuser increases slightly. Therefore, the entropy generation analysis with CFD can be used to reveal the energy loss position of a centrifugal pump for selective optimization. However, the research on entropy production of a multi-stage centrifugal pump is not enough.

Based on the entropy generation method, this paper further studies the complex flow and energy loss of flow passage components in the multi-stage centrifugal pump, analyzes the causes of entropy generation, and provides a theoretical basis for the optimal design of the multi-stage centrifugal pump.

## 2. Entropy Generation Theorem

This study assumes that the temperature of the multi-stage centrifugal pump is constant, thus we only considered the entropy generation rate concerning viscous dissipation.

The transportation equation of specific entropy for single-phase incompressible flow is as follows [29]:

(1) $$\rho \left(\frac{\partial s}{\partial t}+u_{1}\frac{\partial s}{\partial x_{1}}+u_{2}\frac{\partial s}{\partial x_{2}}+u_{3}\frac{\partial s}{\partial x_{3}}\right)=div\left(\frac{\vec{q}}{T}\right)+\frac{\Phi }{T}+\frac{\Phi_{\Theta }}{T^{2}}$$

where $s=\bar{s}+s^{\prime }$, Φ is the viscous dissipation term, and Φ_Θ_ is the entropy production term. 

Equation (1) is changed as follows:

(2) $$\rho \left(\frac{\partial \bar{s}}{\partial t}+\bar{u_{1}}\frac{\partial \bar{s}}{\partial x_{1}}+\bar{u_{2}}\frac{\partial \bar{s}}{\partial x_{2}}+\bar{u_{3}}\frac{\partial \bar{s}}{\partial x_{3}}\right)=\bar{div\left(\frac{\vec{q}}{T}\right)}-\rho \left(\frac{\bar{\partial u_{1}^{\prime }s^{\prime }}}{\partial x_{1}}+\frac{\bar{\partial u_{2}^{\prime }s^{\prime }}}{\partial x_{2}}+\frac{\bar{\partial u_{3}^{\prime }s^{\prime }}}{\partial x_{3}}\right)+\frac{\bar{\Phi }}{T}+\frac{\bar{\Phi_{\Theta }}}{T^{2}}$$

For incompressible flow:

(3) $$\begin{array}{l} \Phi =2\mu \left[{\left(\frac{\partial u_{1}}{\partial x_{1}}\right)}^{2}+{\left(\frac{\partial u_{2}}{\partial x_{2}}\right)}^{2}+{\left(\frac{\partial u_{3}}{\partial x_{3}}\right)}^{2}\right]\\ +\mu \left[{\left(\frac{\partial u_{1}}{\partial x_{2}}+\frac{\partial u_{2}}{\partial x_{1}}\right)}^{2}+{\left(\frac{\partial u_{1}}{\partial x_{3}}+\frac{\partial u_{3}}{\partial x_{1}}\right)}^{2}+{\left(\frac{\partial u_{2}}{\partial x_{3}}+\frac{\partial u_{3}}{\partial x_{2}}\right)}^{2}\right] \end{array}$$

The specific entropy generation rate consists of two parts in turbulent flow: one is the mean term caused by average velocity, the other is the fluctuating term caused by the fluctuating velocity [30]:

(4) $$S_{pro,D}=S_{pro,VD}+S_{pro,TD}$$

(5) $$\begin{array}{l} S_{pro,VD}=2\frac{\mu }{T}\left[{\left(\frac{\partial \bar{u_{1}}}{\partial x_{1}}\right)}^{2}+{\left(\frac{\partial \bar{u_{2}}}{\partial x_{2}}\right)}^{2}+{\left(\frac{\partial \bar{u_{3}}}{\partial x_{3}}\right)}^{2}\right]\\ +\frac{\mu }{T}\left\{{\left(\frac{\partial \bar{u_{1}}}{\partial x_{2}}+\frac{\partial \bar{u_{2}}}{\partial x_{1}}\right)}^{2}+{\left(\frac{\partial \bar{u_{1}}}{\partial x_{3}}+\frac{\partial \bar{u_{3}}}{\partial x_{1}}\right)}^{2}+{\left(\frac{\partial \bar{u_{2}}}{\partial x_{3}}+\frac{\partial \bar{u_{3}}}{\partial x_{2}}\right)}^{2}\right\} \end{array}$$

(6) $$\begin{array}{l} S_{pro,TD}=2\frac{\mu }{T}\left[\bar{{\left(\frac{\partial u_{1}^{\prime }}{\partial x_{1}}\right)}^{2}}+\bar{{\left(\frac{\partial u_{2}^{\prime }}{\partial x_{2}}\right)}^{2}}+\bar{{\left(\frac{\partial u_{3}^{\prime }}{\partial x_{3}}\right)}^{2}}\right]\\ +\frac{\mu }{T}\left\{\bar{{\left(\frac{\partial u_{1}^{\prime }}{\partial x_{2}}+\frac{\partial u_{2}^{\prime }}{\partial x_{1}}\right)}^{2}}+\bar{{\left(\frac{\partial u_{1}^{\prime }}{\partial x_{3}}+\frac{\partial u_{3}^{\prime }}{\partial x_{1}}\right)}^{2}}+\bar{{\left(\frac{\partial u_{2}^{\prime }}{\partial x_{3}}+\frac{\partial u_{3}^{\prime }}{\partial x_{2}}\right)}^{2}}\right\} \end{array}$$

For a numerical analysis, the former can be calculated by the known velocity and temperature, whereas the following is related to the mean temperature and turbulent dissipation rate [31,32]:

(7) $$S_{pro,TD}=\frac{\rho \varepsilon }{T}$$

The total entropy generation rate of different parts can be calculated by the volume integration of each local entropy generation term:

(8) $$\Delta S_{pro}=\Delta S_{pro,VD}+\Delta S_{pro,TD}$$

(9) $$\Delta S_{pro,VD}=\int_{V}S_{pro,VD}dV$$

(10) $$\Delta S_{pro,TD}=\int_{V}S_{pro,TD}dV$$

## 3. Experimental and Numerical Methods

### 3.1. Experimental Model

**Figure 2:**
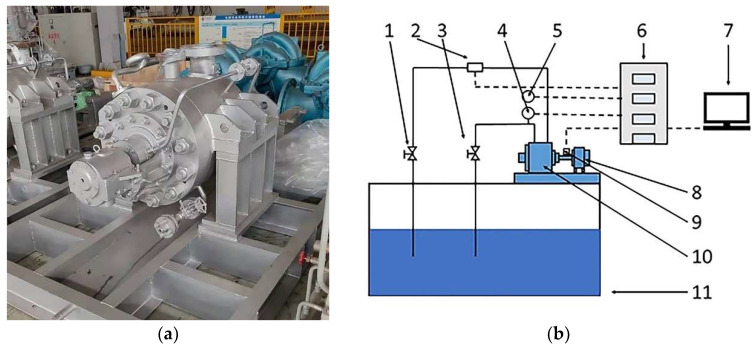
Multi-stage centrifugal pump and its experimental device: (**a**) entity; (**b**) experimental device. 1—outlet pipe valve, 2—electromagnetic flowmeter, 3—inlet pipe valve, 4—inlet pipe pressure transmitter, 5—outlet pipe pressure transmitter, 6—control cabinet, 7—computer, 8—motor, 9—photoelectric tachometer, 10—multi-stage centrifugal pump, 11—reservoir.

A seven-stage centrifugal pump was used as the experimental object, as shown in Figure 2a. Its main performance parameters are shown in Table 1. The main equipment of the experimental device includes the water storage tank, multi-stage centrifugal pump, motor, inlet and outlet pressure sensor, electromagnetic flow meter, photoelectric velocimeter, control cabinet, and computer, as shown in Figure 2b. The models of the main equipment for the performance test are shown in Table 2.

The inlet and outlet of the test device were connected with the reservoir. The inlet and outlet pipelines of the centrifugal pump were equipped with pressure transmitters, the couplings of the pump and motor were equipped with photoelectric tachometers, and the outlet pipelines were equipped with electromagnetic flow meters. The water pump comprehensive performance test system was installed on the computer. During the test, the centrifugal pump was tested for external characteristics within the range of 0 *Q_d_* ~ 1.4 *Q_d_* (1 *Q_d_* = 30 m^3^/h), and the flow was adjusted by the pipe valves.

### 3.2. Grid Generation and Grid Independence Investigation

**Figure 3:**
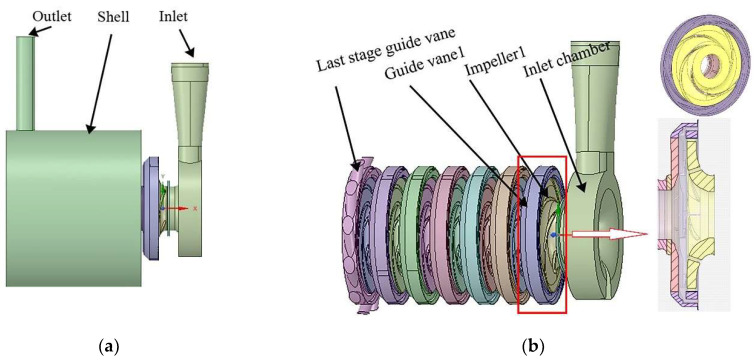
Fluid domain of pump: (**a**) fluid domain; (**b**) the interior of fluid domain assembly.

The original design drawings were three-dimensional and two-dimensional drawings of the whole machine and parts of the BB5 multi-stage centrifugal pump supplied by the pump plant. The main flow passages were the inlet chamber, seven impellers, seven guide vanes, and shell, as shown in Figure 3. The structure and axial section of the pump stage part is shown in Figure 3b.

**Figure 4:**
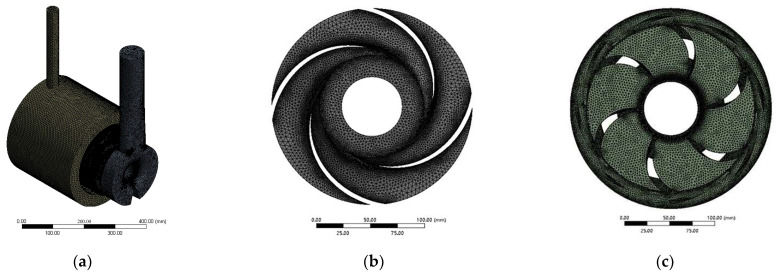
Grids: (**a**) overall grid; (**b**) impeller 2; (**c**) guide vane 2.

Because of the complexity of the physical model, the unstructured tetrahedral grids were adopted in the computational zone, and were created by the software ANSYS Meshing 18.2, as shown in Figure 4. Only the grids of impeller 2 and guide vane 2 are shown due to their similarities. The mesh of the surface with the large curvature and the small gap in the pump were refined with the Curvature and Proximity size functions included in ANSYS Meshing. The grid number of each flow passage component is shown in Table 3. The total number of grids was about 9.83 million. The grid quality was above 0.2 and the average grid quality was about 0.83. The average $y^{+}$ of the key surfaces was reasonable for a safe limit according to the Fluent User’s Guide. Four sets of the grid scheme under the designed flow condition are shown in Table 4. The computational domain keeps proportional similarity to the grid number. The head was selected to assess the impact of the grid number on the final solution. When the total grid number was greater than 9,833,274, the change of the head was little and the change of relative error between the simulated head and test head became smaller. Thus, the total grid number of 9,833,274 was used for the calculation.

### 3.3. Turbulence Model and Boundary Conditions

Considering the large curvature of the internal overflow interface of the centrifugal pump, the governing equations selected in this research were the three-dimensional Reynolds time-averaged N–S equation and RNG k-ε turbulence model [33]. Compared with other turbulence models, the RNG k-ε turbulence is more effective for determining the flow with a high strain rate and large streamline curvature.

The three-dimensional Reynolds-averaged N–S equation is as follows:

(11) $$\frac{\partial {\bar{u}}_{i}}{\partial t}+{\bar{u}}_{j}\frac{\partial {\bar{u}}_{i}}{\partial x_{j}}=-\frac{1}{\rho }\frac{\partial \bar{p}}{\partial x_{i}}+\frac{\partial }{\partial x_{j}}\left(\nu \frac{\partial {\bar{u}}_{i}}{\partial x_{j}}-\bar{u_{i}^{\prime }u_{j}^{\prime }}\right)$$

The RNG k-ε model is identical to the standard k-ε model in form. However, the difference is that the value of the constant is not based on experimental data, but on theoretical analysis. Therefore, in the calculation of complex flows, the RNG k-ε model has higher accuracy [34]. Its equations are as follows:

(12) $$\frac{\partial \left(\rho k\right)}{\partial t}+\frac{\partial \left(\rho ku_{i}\right)}{\partial x_{i}}=\frac{\partial }{\partial x_{j}}\left(\alpha_{k}\mu_{eff}\frac{\partial k}{\partial x_{j}}\right)+G_{k}+G_{b}-\rho \varepsilon -Y_{M}+S_{k}$$

(13) $$\frac{\partial \left(\rho \varepsilon \right)}{\partial t}+\frac{\partial \left(\rho \varepsilon u_{i}\right)}{\partial x_{i}}=\frac{\partial }{\partial x_{j}}\left(\alpha_{\varepsilon }\mu_{eff}\frac{\partial \varepsilon }{\partial x_{j}}\right)+C_{1}\frac{\varepsilon }{k}\left(G_{k}+C_{3}G_{b}\right)-C_{2}\rho \frac{\varepsilon^{2}}{k}-R_{\varepsilon }+S_{\varepsilon }$$

where *C*_1_ = 1.42, *C*_2_ = 1.68, *C*_3_ = 1.39, *α_k_* = *α_ε_* = 0.7179, *μ_eff_* = *μ* + *μ_t_* and *R_ε_* are terms to adapt to the rapid flow with variable rate and streamline curvature, and its expression is:

(14) $$R_{\varepsilon }=\frac{C_{u}\rho \eta^{3}\left(1-\frac{\eta }{\eta_{0}}\right)}{1+\beta \eta^{3}}\frac{\varepsilon^{2}}{k}$$

(15) $$\eta ={\left(E_{ij}\cdot E_{ij}\right)}^{\frac{1}{2}}\frac{k}{\varepsilon }$$

(16) $$E_{ij}=\frac{1}{2}(\frac{\partial u_{i}}{\partial x_{j}}+\frac{\partial u_{j}}{\partial x_{i}})$$

where *C_μ_* = 0.0845, *η*_0_ = 4.38, and *β* = 0.012.

The turbulent flow field of the pump was calculated by ANSYS Fluent 18.2. It was assumed that the flow was steady. The RANS equations were closed with the Reynolds stress turbulence model and standard wall function.

The inlet boundary was set as the pressure inlet according to the actual pressure. Furthermore, the hydraulic diameter was set at 40 mm and the turbulence intensity was set at 5%. The mass flow outlet was set as the outlet boundary.

All overflow areas were set as the fluid domain, and the fluid was liquid water. The impeller area was set as the rotating basin in the rotating reference system, where the rotating speed was 2980 rpm (note that the rotating direction of the impeller follows the right-hand rule), and the inlet section, guide vanes, middle section, and outlet section were the static areas.

In addition to the inlet and outlet interfaces, all walls were set as the no-slip boundary. The near-wall region was solved by the standard wall function.

The SIMPLE algorithm was used to couple the pressure and velocity. A residual of 1 × 10^−4^ was set as the convergence criteria for the numerical analysis.

### 3.4. Validation of the Numerical Results

This study calculated the hydraulic performance under different flow conditions from 0.6 *Q_d_* to 1.4 *Q_d_* (1 *Q_d_* = 30 m^3^/h). The corresponding equations of head, shaft power, and efficiency are as follows:

(17) $$H=\frac{p_{out}-p_{in}}{\rho g}$$

(18) N=Mnπ/30

(19) $$\eta =\eta_{h}\eta_{m}\eta_{v}$$

where *η_h_*, *η_m_*, and *η_v_* are hydraulic efficiency, mechanical efficiency, and volumetric efficiency, respectively. Their formulas and empirical expressions are as follows [35]:

(20) $$\eta_{h}=\frac{\rho gQH}{M\omega }$$

(21) $$\eta_{m}=1-0.07{\left(0.01n_{s}\right)}^{-1.18}$$

(22) $$\eta_{v}=1-0.028{\left(0.01n_{s}\right)}^{-0.6}$$

**Figure 5:**
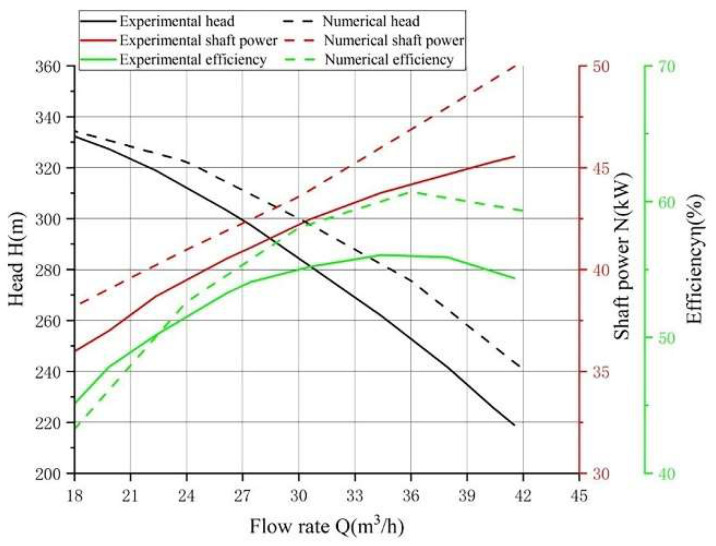
Comparison of numerical and experimental results.

The main flow field simulation results at 2980 rpm were plotted to obtain the curves of *H-Q*, *N-Q*, and *η-Q* and were compared with the experimental results, as shown in Figure 5. The simulated head, shaft power, and efficiency are basically the same as the experimental results. Under the design condition (*Q* = 30 m^3^/h), the head error is 6.7%, the shaft power error is 5.7%, and the efficient error is 5.2%. The pump efficiency is the product of hydraulic efficiency, mechanical efficiency, and volumetric efficiency, as shown in Equation (13), but only hydraulic losses were used in the determination of the simulated pump efficiency; thus, there is a deviation between the simulation and the experimental results. When the flow rate is greater than 36 m^3^/h, the error increases gradually. The reason is that the compressibility of the system increases as the high-speed rotating fluid speed in the multi-stage centrifugal pump increases. The compressibility of fluid in the multi-stage centrifugal pump has a certain impact on the mechanical loss and volume loss of the pump. In addition, air bubbles generated by cavitation also cause energy loss of the pump. However, only the incompressible system was considered and the cavitation effect of the pump was also not considered in this paper, thus, the deviation was larger. However, the deviation was still within 10%. These results show that the simulation results agree well with the experimental results.

## 4. Results and Discussion

### 4.1. Entropy Generation of Different Components under the Rated Flow Condition

**Figure 6:**
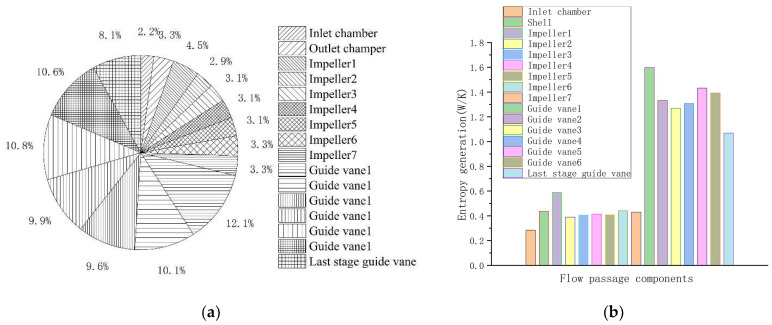
Entropy generation of each channel component under the rated flow: (**a**) entropy generation ratio; (**b**) entropy generation value.

The entropy generation of each channel component under the rated flow condition (30 m^3^/h) is shown in Figure 6. Figure 6a shows that the guide vanes of the centrifugal pump are the primary areas of entropy generation, accounting for 71.2%. Entropy generation of guide vane 1 to guide vane 6 is roughly the same, and entropy generation of the last-stage guide vane is relatively low compared with others because there are no middle components in the last-stage guide vane. Entropy generation of the inlet chamber, shell, and impeller is roughly the same. Impellers account for 23.3% of the total entropy generation and impeller 1 has the highest entropy generation. It is suggested that the primary reason for energy loss is the guide vanes, followed by the impellers. Figure 6b shows that entropy generation of the guide vanes is about 9 W/K. Guide vane 1 has the largest entropy generation value, which is about 1.6 W/K. Additionally, the average entropy generation of the impellers is about 0.5 W/K, but its total value cannot be ignored.

**Figure 7:**
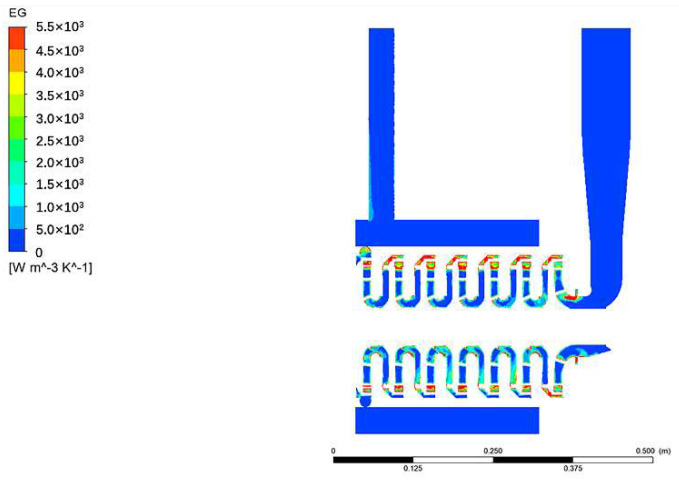
Entropy generation distribution of axial section at rated flow condition.

Figure 7 displays the entropy generation distribution of the axial section under the rated flow condition. The figure suggests that the entropy generation is mainly concentrated in the impeller inlet, impeller outlet, and positive guide vane, which suggests the hydraulic loss mainly occurs in these places and the entropy generation method is helpful to accurately calculate the location and size of the energy loss.

### 4.2. Entropy Generation of Different Components under Different Flow Conditions

**Figure 8:**
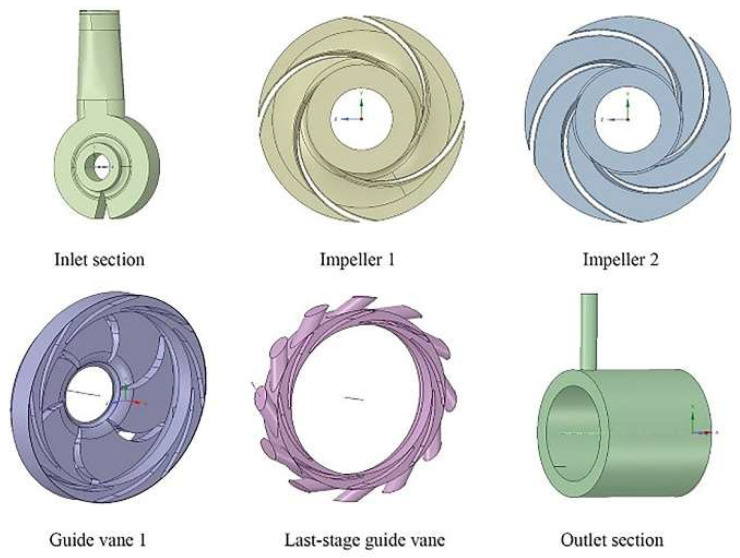
The typical overcurrent parts.

The typical overcurrent parts of a multi-stage centrifugal pump include the inlet chamber, outlet chamber, impellers, and guide vanes, which are shown in Figure 8. The results of impellers 3–6 and guide vanes 2–6 are not shown because there are similarities in their structures.

**Figure 9:**
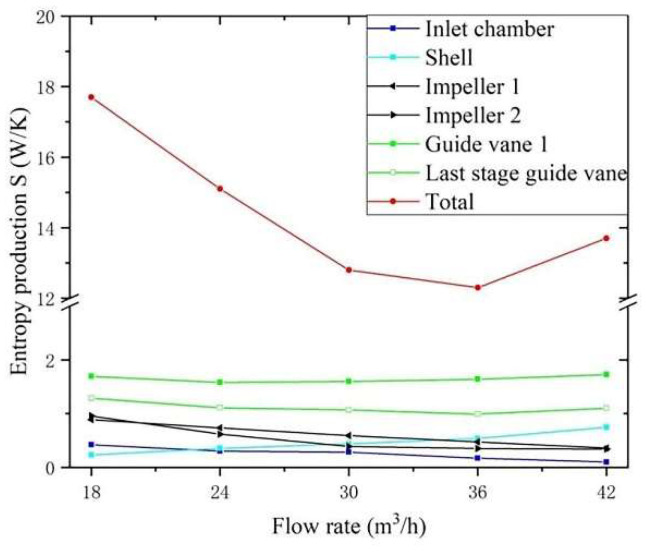
Entropy generation under different flow conditions.

Figure 9 displays the entropy generation of typical overcurrent parts under different flow conditions (0.6 *Q_d_* ~ 1.4 *Q_d_*). Firstly, the total entropy generation first descends and then ascends as flow rate increases. The minimum value is achieved at 1.1 *Q_d_*, which is opposite to the variation trend of the efficiency curve in the characteristic curve. This shows that there is a correlation between entropy generation and efficiency of the centrifugal pump. The smaller the entropy production, the higher the efficiency. Secondly, guide vanes have the largest entropy generation among all overcurrent parts. Additionally, their change is alleviation under different flow conditions. When the flow rate is greater than 1.1 *Q_d_*, the entropy production increases slightly. It shows that the energy loss of the guide vane cannot be reduced by simply changing the flow conditions, and it needs to be optimized. Besides, entropy generation of impeller 1 and impeller 2 descends gradually as the flow rate increases. It illustrates that the bigger the flow rate, the smaller the entropy generation in impellers, but it also means an increase in energy costs. Additionally, the entropy generation in the inlet chamber decreases with the increase in flow, and increases in the outlet chamber. The variation amplitude of the outlet chamber is larger than that of other overcurrent parts.

### 4.3. Entropy Generation of the Impellers and Guide Vanes

Only the results of impeller 2 and guide vane 2 were analyzed because there are similarities in the entropy generation distribution of all impellers and guide vanes.

**Figure 10:**
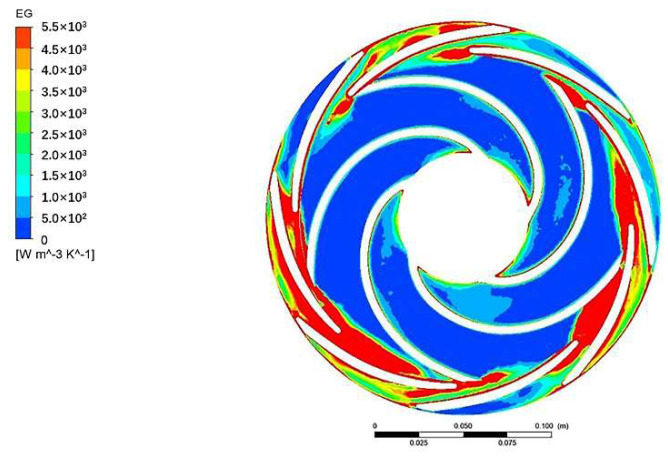
Entropy generation distribution of impeller 2 and guide vane 2 in the middle section.

Figure 10 displays the entropy generation of impeller 2 and guide vane 2 in the middle section. On the whole, the entropy generation of the impeller is distributed in a central symmetry. Additionally, the impeller inlet, guide vane tongue, and the channel of the guide vane have relatively more entropy generation compared with others.

**Figure 11:**
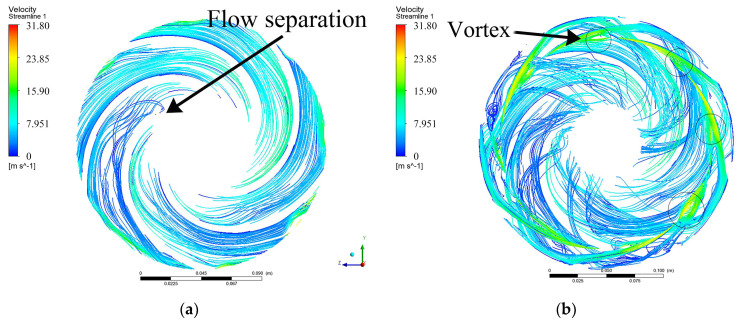
Streamlines of impeller 2 and guide vane 2 in the middle section: (**a**) impeller 2; (**b**) guide vane 2.

Figure 11 displays the streamlines of impeller 2 and guide vane 2. It can be observed that the streamlines in the impeller are smoother compared with the guide vane. The centrifugal pumps add mechanical work to fluid through the impellers and convert it into pressure energy. The flow along the channel is subject to an adverse pressure gradient, thus flow separations occur easily, which is one of the reasons resulting in entropy generation. The high-speed rotating fluid in the impeller is discharged from the outlet and enters the diffuser of the guide vane. The flow area increases, the flow rate decreases, and some kinetic energy is converted into static energy, which further increases the fluid pressure. When the impeller outlet section rotates near the guide vane tongue, the flow rate decreases because of the impeller pushing action and the reduction in the flow area, resulting in vortices and also resulting in entropy generation.

**Figure 12:**
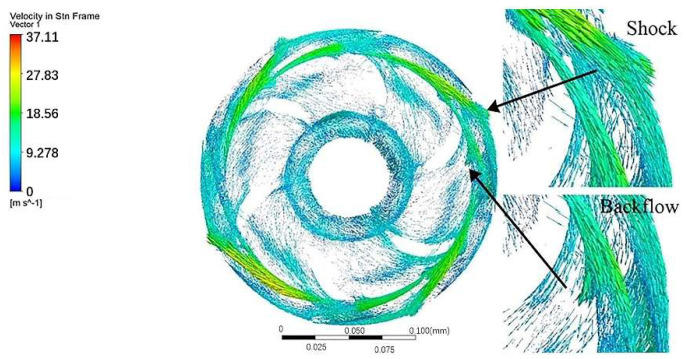
Velocity vectors of guide vane 2 in the middle section.

Figure 12 displays the velocity vectors of guide vane 2 in the middle section. The fluid at the impeller outlet enters the positive guide vane, then passes through the reverse guide vane, and finally flows to the next-stage impeller. Due to the diffuser deceleration, the flow velocity of the reverse guide vane is lower compared with the positive guide vane. Moreover, the partial, enlarged view of the upper left corner suggests that the fluid enters the guide vane area with a larger flow area and impacts the wall surface, which changes that part of the kinetic energy into heat energy and static pressure, resulting in energy loss. The partial, enlarged view of the lower left corner suggests that the fluid reflux at the guide vane diaphragm tongue enters the impeller when the fluid enters the guide vane, resulting in energy loss.

**Figure 13:**
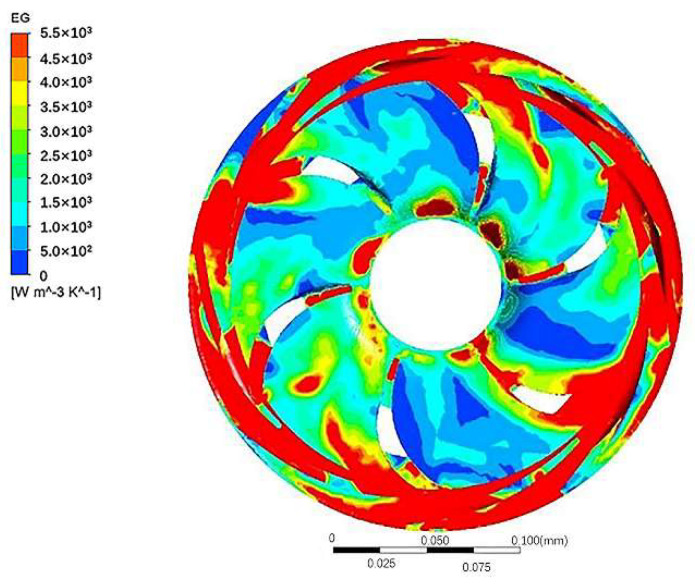
Entropy generation distribution of the blade of impeller 2.

Figure 13 displays the entropy generation distribution of guide vane 2. It shows that the forward guide vane has large entropy generation and it is the primary part where energy loss occurs. The space of the area becomes larger because the high-speed fluid flows from the impeller outlet into the guide vane, which changes the fluid kinetic energy into heat energy and static pressure. The fluid impacts the outer edge of the guide vane, resulting in an increase in entropy generation. In addition, there is the reflux near the guide vane tongue which also increases the energy loss. This corresponds to the information of the velocity vector in Figure 11.

**Figure 14:**
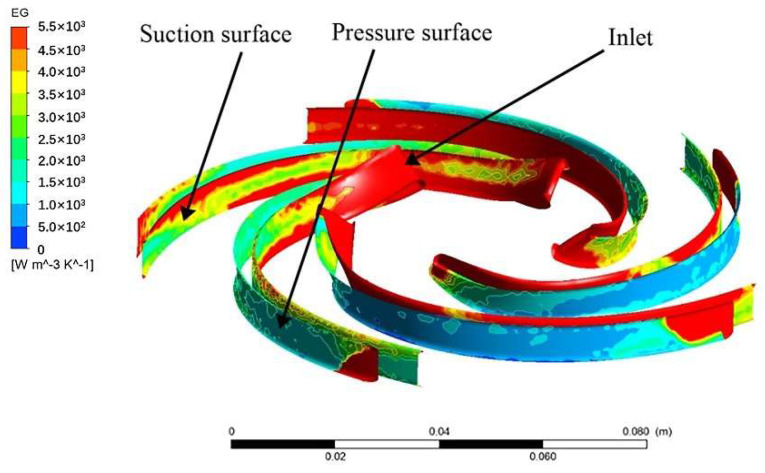
Entropy generation distribution of the blade of impeller 2.

Figure 14 displays the entropy generation distribution of the blade of impeller 2. It shows that the entropy generation of the blade pressure surface is smaller compared with the suction surface because there is flow separation at the suction surface. The entropy generation of the impeller inlet area and outlet area is larger due to vortex and flow separation.

## 5. Conclusions

A BB5 multi-stage centrifugal pump was investigated by numerical analysis using the entropy generation method with the RNG k-ε turbulence model, and its energy loss characteristics were studied. The simulation was performed at the flow rate of 0.6 *Q_d_* ~ 1.4 *Q_d_* (1 *Q_d_* = 30 m^3^/h). Additionally, the accuracy of the numerical simulation was verified with the performance comparison of the numerical results and experimental results.

The main conclusions are as follows:

- Entropy generation of the guide vanes and impellers accounted for 71.2% and 23.3% of the total entropy generation at a flow rate of 1.0 *Q_d_*, respectively. The entropy generation of the guide vanes is the biggest entropy generation at about 9 W/K, thus the guide vanes are the main hydraulic loss domains, followed by impellers.
- There are many vortices at the guide vane inlet, which are located on the tongue of the guide vane. Additionally, there are flow separations in the impeller. The fluid impacts the outer edge of the guide vane, which leads to the increase in entropy generation. Furthermore, there is the reflux near the guide vane tongue, which also increases entropy generation.
- The entropy generation of the positive guide vane and blade suction surface is greater compared with the reverse guide vane and pressure surface, respectively.

## Figures and Tables

**Table 1:** Main performance parameters of multi-stage centrifugal pump.

Rated Flow (m^3^/h)	Rated Speed (rpm)	Rated Head (m)	Cavitation Allowance (m)	Shaft Power (kw)	Motor Power (kw)
30	2980	252.2	30	30	30

**Table 2:** The models of main equipment for performance test.

Electromagnetic Flowmeter	Resonant Pressure Transmitter	Motor	Photoelectric Tachometer	Multi-Stage Centrifugal Pump
E-mag	EJA430E	Variable-Frequency Adjustable-Speed Three-Phase Roller	Laser Doppler Velocimeter	BB5 Seven-Stage Pump

**Table 3:** Grid number and the average *y*^+^ of the surfaces for each flow passage component.

Region	Grids	*y* ^+^	Region	Grids	*y* ^+^
Inlet chamber	564,800	90	Guide vane 1	806,900	125
Impeller 1	437,800	144	Guide vane 2	807,000	132
Impeller 2	504,700	122	Guide vane 3	811,200	120
Impeller 3	504,900	121	Guide vane 4	807,000	120
Impeller 4	502,500	123	Guide vane 5	809,800	120
Impeller 5	507,200	123	Guide vane 6	808,700	124
Impeller 6	503,900	123	Last-stage guide vane	653,400	124
Impeller 7	510,000	125	Shell	293,500	180

**Table 4:** Grid independence verification.

Total Number of Grids	Head (m)	Relative Error with Experimental Data (%)
7,535,621	320.6	12.1
8,904,698	310.7	9.2
9,833,274	300.2	6.7
11,015,236	298.3	5.5

## Data Availability

Not applicable.
